# Regorafenib adjusted dose for Chilean patients with chemoresistant metastatic colorectal cancer: a case series

**DOI:** 10.3332/ecancer.2018.875

**Published:** 2018-10-02

**Authors:** José Luis Leal, Juan Briones, María Elisa Herrera, Bettina Müller, Bruno Nervi, Sebastián Mondaca

**Affiliations:** 1 Medical Oncology Responsability Centre, Instituto Nacional del Cáncer, Santiago 8380455, Chile; 2 Medical Oncology Service, Instituto Oncológico Fundación Arturo López Pérez, Santiago 7501066, Chile; 3 Hemato-Oncology Department, Complejo Asistencial Sótero del Río, Puente Alto, Santiago 8207257, Chile; 4 Hemato-Oncology Department, Pontificia Universidad Católica de Chile, Santiago 8330032, Chile; 5 Hemato-Oncology Department, Hospital Base de Valdivia, Valdivia 5090146, Chile

**Keywords:** regorafenib, antiangiogenic, colorectal neoplasms, Latin America

## Abstract

**Background:**

Regorafenib is a therapeutic alternative for patients with metastatic colorectal cancer (MCRC) resistant to conventional therapies. The reported toxicity is relevant and there is no data on Latin American patients. The objective was to evaluate the overall survival (OS), progression-free survival (PFS) and quality of life (QoL) in a prospective cohort of Latin American patients treated with an adjusted dose of regorafenib.

**Methods:**

We prospectively recruited patients with MCRC that progressed to standard therapy. A dose escalation algorithm was used. OS, PFS, response rate and QoL were evaluated.

**Results:**

We recruited 13 patients between June and November 2015. The median age was 60 years. Median OS was 8.6 months and median PFS was 2.2 months. The response rate was 8%. Grade 3–4 toxicities included grade 3 palmoplantar erythrodysesthesia in 23% and grade 3 fatigue in 12% of patients.

**Conclusion:**

Regorafenib treatment is effective in Latin American patients with conventional therapy resistant MCRC.

## Background

Colorectal cancer (CRC) is a common disease, being the second most frequent cancer type in women and the third in men worldwide [1]. In the past decade, we have observed a significant improvement in clinical outcomes associated with the development of new treatment strategies for metastatic CRC, and median survival has been prolonged from 6 months to over 30 months, with the sequential use of conventional chemotherapy (CT) including fluorouracil, irinotecan, oxaliplatin, in combination with monoclonal antibodies such as bevacizumab, cetuximab or panitumumab [2, 3].

Many patients maintain a good performance status until progression with or after these therapies and are candidates for other treatments, but options are limited, which is why an unmet need exists. Many trials have examined the role of cytotoxic CT for resistant to standard therapies metastatic CRC patients [4] with discrete results. The RECOURSE trial demonstrated the activity of TAS-102, a combination of triflouridine and tipiracil, compared to placebo, showing benefit in OS [5]. Regorafenib is an orally administered intracellular multi-kinase inhibitor that binds to several intracellular kinases, with a strong effect against vascular endothelial growth factor receptors (VEGFR) 1 to 3, platelet derived growth factor receptor (PDGFRB), fibroblast growth factor receptor 1 (FGFR1) and oncogenic mutant kinases KIT, RET and BRAF [6]. The reported toxicity associated with this drug is relevant, which raises doubts concerning the currently approved dose [7], thus the benefit of an adjusted dose is being evaluated [8].

Regorafenib is a standard treatment option in chemoresistant metastatic CRC based on two phase III trials [9, 10], and is recommended in treatment guidelines [11]. However, the Latin American population has not been represented in either of these studies. Therefore, we cannot rule out the possibility of there being a difference in effectiveness as well as in tolerance to this drug in this population. Interactions have been reported between ethnicities and these variables [12, 13].

The objective of this series was to evaluate the OS, progression-free survival (PFS) and quality of life (QoL) of regorafenib in a cohort of Latin American patients following at least two lines of systemic treatment.

## Methods

### Patients

A prospective cohort was designed, in which patients treated at two hospitals in Chile were enrolled between June and November 2015.

Patients had to be over 18 years, diagnosis of CRC by biopsy, Eastern Cooperative Oncology Group Performance Status (ECOG-PS) 0 or 1 and metastatic measurable disease by RECIST 1.1 [14]. They must have received two or more lines of CT including: 5-fluorouracil and/or capecitabine, oxaliplatin and irinotecan. We excluded patients with an estimated life expectancy below 3 months, and significant cardiac, hepatic or renal dysfunction. All patients signed a consent form that was approved by the institutional ethics committee.

### Procedures

Treatment with regorafenib was administered once daily from days 1 to 21 of each 28-day cycle until disease progression, death, unacceptable toxic effects or withdrawal of consent. The initial dose was of 80 mg and was re-evaluated in 2 weeks. The dose was increased to 120 mg/day in case of none or mild toxicity. All patients received palliative care at the physician’s discretion.

### Evaluations

Tumour response evaluation was performed by computerised axial tomography using RECIST version 1.1 criteria every 8 weeks during treatment. We assessed patient-reported outcomes using the EORTC-QLQ-C30 questionnaire [15], performed at baseline and every 4 weeks before each cycle. Patients were followed-up every 2 weeks during the first 2 cycles, and then every 4 weeks. Adverse events were registered using the NCI CTCAE manual, 4.0 version [16].

### Statistical analysis

A descriptive analysis of the population was performed using central tendency and dispersion measures, according to the nature of the variable. Student’s *T*-test was used on paired samples to compare continuous variables. Kaplan–Meier method was applied in order to evaluate OS and PFS.

To evaluate QoL, we performed a linear transformation of the EORTC QLQ-C30 questionnaire into a 0–100 score [17]. For a given score, a decrease of more than 10 points without a successive recovery was considered to be significant [18]. GraphPad Prism 7.0 software was used for the statistical analysis.

## Results

### Patients

We enrolled 13 patients in this series during the study period, 6 (46.1%) were male and 7 (53.9%) female. The median age of patients was 60 years (range: 43–76) and 8 (61%) had ECOG 1. The most common primary site was the colon in 77% of patients. In this cohort, only six patients had a RAS study and of these, two had a gene mutation. Forty-six percent of patients had received more than two lines of CT. There were no patients previously treated with biological therapies. Patient’s main characteristics are summarised in Table 1.

### Response and survival

**Figure 1.**
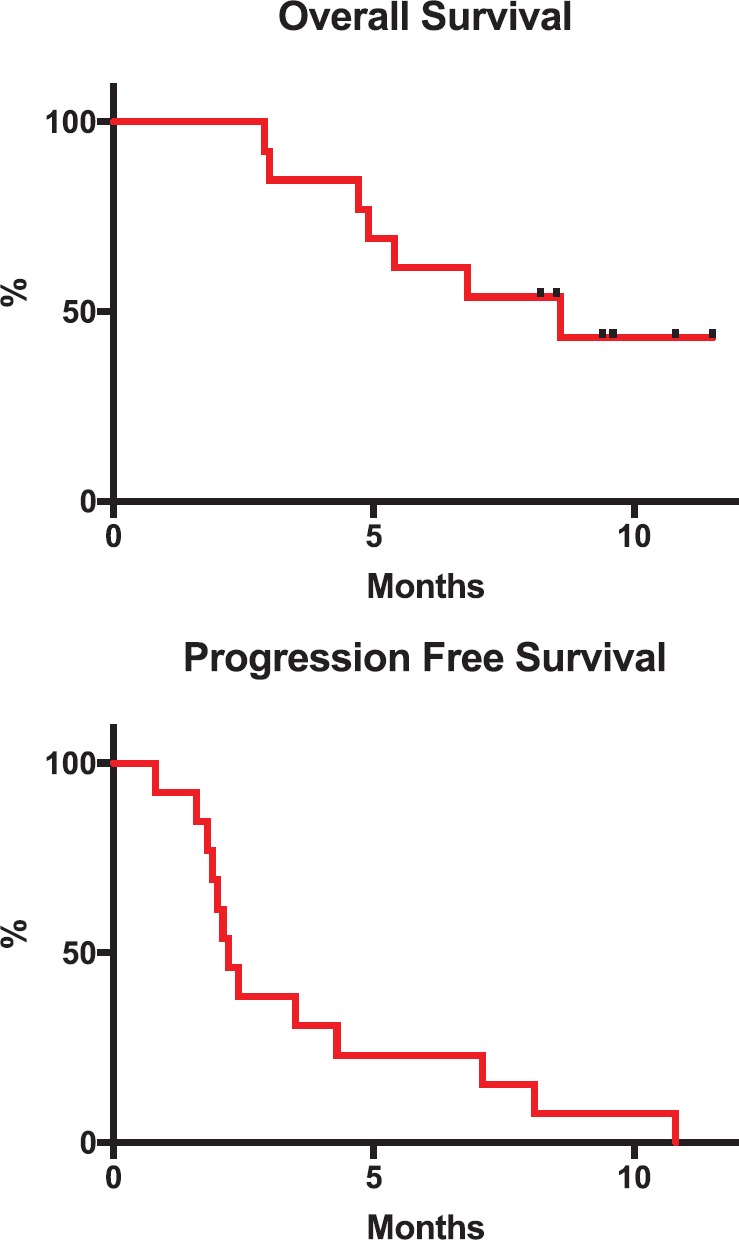
Kaplan Meier plot of OS and PFS.

The average dose of regorafenib was of 95.5 mg. The average number of cycles was 4.1 (range: 1–9, standard deviation 4.1 ± 2.619) and the median follow-up time was 5.4 months. One patient (8%) had a partial response and three patients (23%) had stable disease. All patients were included in the survival analysis. Median PFS and OS were 2.2 and 8.6 months, respectively (Figure 1). Survival at 6 months was 58%. At the time of analysis, 11 patients had suspended treatment due to progression and 1 due to toxicity (grade 3 fatigue).

### Toxicity and quality of life

The most frequent grade 3–4 toxicity was grade 3 palmoplantar erythrodysesthesia (23%). There was neither grade 4 toxicity nor treatment-related deaths (Table 2). Average weight at the beginning and end of treatment were 70.4 kg and 65.3 (*P* = 0.006), respectively. The median time to QoL definitive deterioration was 3 months.

## Discussion

Our series suggests that regorafenib is effective and tolerable in Latin American patients with metastatic CRC (MCRC) previously treated with cytotoxic CT. In this study, regorafenib showed a discrete activity and an acceptable toxicity, which is consistent with previous results [9, 10]. The CORRECT study included patients with resistant metastatic CRC that had received all standard therapies. In the group treated with regorafenib, the median OS was 6.4 months and the PFS was 1.9 months; the objective response rate was 10%. In our series, the OS was slightly better, reaching a median of 8.6 months. This may be explained because, unlike CORRECT, our patients had not received any previous biological therapies including antiangiogenics, which is one of the action mechanisms of regorafenib, or epithelial growth factor (EGFR) inhibitors in cases with wild-type RAS. In Chile, as in other countries with limited resources, these drugs are not covered by the public healthcare system. In CONCUR study, strictly an Asian population, 40% of patients had not received previous biological treatment and OS and PFS were 8.8 and 3.2 months, respectively.

Our data showed low-grade 3–4 toxicity: grade 3 hand-foot syndrome (23%) and grade 3 fatigue (15%). This may be due to the protocol we used, in which the maximum standard dose of 160 mg/day was not reached. In the CORRECT study, 58% of patients developed grade 3 or 4 toxicity and 38% needed dose reduction [9]. In one phase III-B study, 54% of patients reported grade 3 adverse events [19].

QoL results in our cohort are concordant with the analysis performed by the pivotal studies, in which there is no evidence of QoL degradation. The plateau in the QoL domain curve represents the subgroup that maintains the treatment due to a clinical benefit. The main limitation of this series is its low number of patients, which precludes the obtainment of precise results. However, we consider that our experience adds new knowledge of the effectiveness and toxicity of regorafenib in Latin American population. We also used a lower dose regimen, which could aid in the issue of cost-effectiveness [20] that is of particular importance for developing countries. In a previous report from a low-resource setting, patients with chemoresistant MCRC received standard dose regorafenib in a cancer centre in India. This study showed similar outcomes in terms of efficacy and toxicity than pivotal phase III trials [21]. In our series, the toxicity seemed lower while maintaining similar efficacy, suggesting that a dose escalation strategy might be a reasonable alternative.

## Conclusions

Our data suggest that Regorafenib is an effective treatment for Latin American patients with chemoresistant MCRC, with an acceptable toxicity using an adaptive dose escalation schedule. This must be confirmed in larger analytical studies, but might represent a more attractive alternative in terms of safety and cost-effectiveness.

## Funding statement

This research did not receive any specific grant from funding agencies in the public, commercial or not-for-profit sectors.

## Conflicts of interest

The authors have no conflicts of interest to declare.

## Figures and Tables

**Table 1.** Basal characteristics of the study population.

Characteristic	Number (%)
Median age (range, year)	60 (43–76)
Gender Male Female	6 (46) 7 (54)
ECOG PS 0 1	5 (38) 8 (61)
Primary Site Colon Rectum	10 (77) 3 (23)
Histology Adenocarcinoma Mucinous Other	11 (85) 1 (7,5) 1 (7,5)
RAS Mutation No Yes Unknown	4 (30) 2 (15) 7 (55)
Stage at Debut I–III IV	3 (23) 10 (77)
Previous CT number 2 >2	7 (54) 6 (46)
Previous use of biological therapy Yes No	0 (0) 13 (100)

CT: Chemotherapy

**Table 2.** Treatment-related adverse events of the study population.

Toxicity type	Grade 3 N (%)	Grade 4 N (%
Hematologic	-	-
Diarrhea	-	-
Palmoplantar erythrodysesthesia	3 (23%)	-
Cardiovascular (hypertension)	1 (7%)	-
Fatigue	2 (15%)	-
